# Determining what matters: data resources for examining maternal health equity

**DOI:** 10.3389/fpubh.2025.1499468

**Published:** 2025-02-28

**Authors:** Leremy A. Colf, Karina M. Shreffler

**Affiliations:** Fran and Earl Ziegler College of Nursing, University of Oklahoma Health Sciences Center, Oklahoma City, OK, United States

**Keywords:** maternal health, linked data, health equity, birth outcomes, health policy

## Abstract

Maternal morbidity and mortality (MMM) rates in the U.S. are high and increasing, and are disproportionately experienced by understudied, underrepresented, and underreported (U3) populations, especially Black, Indigenous, and/or rural women. Decreasing MMM among U3 women would substantially improve maternal health equity and health outcomes, yet current data limitations inhibit our ability to fully understand the reasons underlying the disparities or regional nuances. This article calls for leveraging diverse, publicly available data such as deidentified health system utilization data; geocoded locations of hospitals providing multiple levels of maternal care services; and social determinants and demographic data into a series of linked datasets to enable county-level investigations of maternal health equity, healthcare utilization, and health outcomes.

## Introduction

Maternal morbidity and mortality (MMM) rates in the U.S. are higher than in all other industrialized nations and are increasing (1, 2). In 2021, ~1,205 women died of causes related to pregnancy in the U.S. (3), a 28.5% increase from the previous year (4) and a 37.4% increase since 2019 (5). Severe maternal morbidity (SMM) is substantially (50–100 times) more common than maternal mortality (6) and is also increasing, affecting 50,000+ women each year, with understudied, underrepresented, and underreported (U3) populations experiencing the worst outcomes (7). Black and Indigenous women, for example, are three to four times as likely as non-Hispanic White women to experience SMM, with Indigenous women living in rural areas at highest risk for adverse birth outcomes (8). The disproportionately high rates of MMM experienced among Black and Indigenous women reflect persistent patterns, due to in part to systemic factors including discrimination, segregation, and historical laws that have resulted in the accumulation of disadvantages across generations (9). Black and Indigenous women have elevated prevalence rates of chronic conditions associated with MMM (10), and they are more likely to give birth in lower performing hospitals (11). Women living in rural areas had a 9% higher probability of SMM and mortality than urban residents in the U.S. between 2007 and 2015 (12). Increased risks for those living in rural areas arise from numerous factors, including lack of access to maternal care services, long travel times, and general social and economic vulnerability (13, 14). Rather than improving over time, increasing numbers of rural hospitals have recently eliminated maternal care services or closed entirely, potentially worsening the situation (15, 16).

Despite the clear health impacts of these risks on both mothers and their infants, little research exists on the effects of geographic barriers to care in the U.S., especially for U3 populations. Addressing this problem will require combining deep expertise in two very different technical areas: maternal health vs. data science. Additional technical hurdles must be overcome on the data side, as current data limitations reduce the ability to investigate geographic barriers for maternal care utilization. While data on relevant variables (i.e., demographics, socioeconomics, and maternal care utilization) are all publicly available, these data are located in disparate datasets, lack common definitions and infrastructure, are technically challenging to link, and require extensive methods development to use for research. Common data elements and formats are necessary for the differential comparison of U3 and majority populations, as well as for evaluation of the impact of interventions (17). However, data scientists collecting, cleaning, and compiling the relevant datasets is not enough; maternal health researchers must be part of the data development process to ensure the datasets match the most important research questions; additionally, maternal health researchers must be trained on how to effectively use the data. Although there are challenges to developing a national maternal care database, it would be immensely valuable in the identification of geographic barriers to care and the development of evidence-based interventions to advance maternal health equity. Only together can researchers and practitioners develop, implement, and evaluate strategies that will improve maternal and infant outcomes for U3 populations.

### Geographic barriers to maternal care

Access to perinatal care is a well-documented need for pregnant and postpartum women, particularly those living in rural communities. Pregnant patients in rural communities have less access to clinical care in their communities and are more likely to have inadequate transportation options to access obstetric services (1). They are also less likely to have access to obstetric specialists in their communities (18). Obstetric services in rural communities are at increased risk for closure due a variety of factors such as reimbursement difficulties, declining patient volumes, and staffing difficulties (19). Recent closures of obstetric facilities resulted in more than 50% of all rural counties in the U.S. being without obstetric services (20). As closures of maternity care facilities in the U.S. continue to increase, it is essential to extend our knowledge to be able to intervene effectively to improve health outcomes.

Previous studies, largely conducted outside the U.S., have examined the relationship between geographic distance/driving time and adverse outcomes in pregnancy, with mixed results and outcomes due to limited data and a high number of complicating factors [see (21–25)]. In general, travel times >20–30 min are associated with adverse outcomes (22, 23). Further, the rate of out-of-hospital births is more than double for expectant women living 30+ km (18.6 miles) from care, particularly for U3 women (26).

### A call for linked data on maternal care

The Netherlands has a national perinatal registry (Netherlands Perinatal Registry or Stichting Perinatale Registratie Nederland) with uniform data collected each year on locations and outcomes in perinatal care. Utilizing this registry, researchers were able to determine that travel times to maternal care of >20 min resulted in increased mortality and adverse outcomes (23). Using this registry and associated research, a national steering committee on perinatal care identified geographic barriers to care as a major contributor to poor maternal child outcomes in the country. They created a nation-wide stakeholder advisory group and conducted listening sessions. They then developed evidence-based interventions and used health-policy solutions to improve perinatal outcomes. Examples of interventions included: auditing perinatal deaths in term babies, instituting prenatal screening for congenital anomalies, and establishing a commission on perinatal care. They also proposed assigning a case manager to every pregnancy, developing birth plans with all expectant women, and instituting mandatory house visits. Maternal and child outcomes improved dramatically nationwide after implementing these interventions (27, 28). This clearly demonstrates the immense value of a national maternal care dataset.

In comparison to the Netherlands, the U.S. has almost 20-fold higher maternal mortality rates and nearly 50-fold higher rates for certain racial/ethnic groups (29). The U.S. is in dire need of evidence-based interventions in maternal care, yet we lack the national datasets necessary for researchers to generate that evidence. A handful of U.S. studies have measured the driving distance to maternal care services, but they do not provide underlying data for other researchers to use, so their ability to inform the study of geographic barriers to care among U3 populations is unclear [see (14, 30, 31)]. A national dataset that incorporates geographic barriers to maternal care with a special focus on U3 women would enable research, policy, and healthcare communities to identify needs and implement interventions to advance maternal health equity.

There are multiple reasons why such a dataset can be critically needed but not yet exist in the U.S. Of note, single-payer or universal healthcare systems like those seen in the Netherlands or the U.K. enable these national datasets by default, whereas the fragmentation of the U.S. healthcare system makes the creation of a dataset much more challenging. First, it is difficult to find and link data on health outcomes, disparities/inequities, and U3 populations. The Federal government, the source of the majority of these data, has been directed where possible to make data publicly available under the OPEN Government Data Act (32). While this would make a wealth of data available and easy to find and use, no funds were allocated to accomplish this work. Second, creating and sharing datasets is of great benefit to other researchers, but limited benefit to those owning/linking/sharing the data, so they are disincentivized to share those data (33). Third, creating and linking these datasets requires technical expertise in data management, big data analysis, medical diagnosis and billing, social determinants of health, geographic information systems and geocoded data, and health equity, which are highly varied skillsets. The technical skills in particular are a substantial barrier to creating and disseminating high-quality, easily accessible data sources (34). This undertaking would require the building and sharing of a highly versatile dataset in easily accessible formats to allow researchers to analyze and compare health equity and geographic barriers to care across U3 populations and racial, social, and economic underlying factors.

### Data challenges

Building a comprehensive dataset will require leveraging diverse, publicly available data such as deidentified electronic health record data; geocoded locations of hospitals providing multiple levels of maternal care services; and social determinants and demographic data at the county-level. While individual research teams can collect these data locally, HIPAA, PII, and data sharing restrictions render the creation of a national dataset nearly impossible. Combining the home geographic region, demographic/socioeconomic information of perinatal patients, and maternal and child health outcomes, would likely enable identification of individuals and violate HIPAA. In addition, health data have become a big business. Health data are estimated to be worth $300–$450 billion per year, creating strong incentives against sharing data or making data interoperable for other researchers to use, analyze, or combine (35, 36). This results in small, fragmented, expensive datasets and/or strong restrictions on what data can be used for research and limiting the applicability of potential results. This paradigm extends to maternal health data: multiple researchers have examined maternity care deserts with informative analyses, but none shared the underlying data. In addition, existing reports focus on the availability of facilities (i.e., maternal care deserts) as a measure of health equity rather than the ability to use those facilities (i.e., health outcomes). For example, the March of Dimes Maternity Care Deserts report (37) ranks each county across the U.S. by the number of facilities in that county. However, while researchers and policymakers can reference these reports, they cannot conduct additional research for other county level measures or moderate by population characteristics.

### New opportunities for data linking

Due to these many restrictions and limitations, it becomes necessary to collect even larger, aggregate datasets and use the resultant increased statistical power to conduct rigorous population-level research rather than individual-level research. Utilizing this approach enables the use of numerous national aggregate data sources. These data sources have standardized reporting, multi-year collection, and rigorous methods that are already understood and accepted by the broader research community. While this approach is challenging, utilizing national datasets (with reporting at the county level) means that for the first time, it is possible in the U.S. There are numerous potential sources of publicly available data that could be utilized for this purpose. For example, the Healthcare Cost and Utilization Project (HCUP) collects discharge data on hospital inpatient visits across the U.S. (38). These data are de-identified and made publicly available through a dashboard. Data can be accessed at the county level (for participating states) and stratified by aggregated, clinically-meaningful categories (Clinical Classifications Software Refined, or CCSR). The publicly available data include 15 CCSR codes related to pregnancy (see Table 1). The CCSR groups in the dataset can easily be binned into routine vs. high-acuity visits, defined as those visits likely to result in SMM. Socio-demographic variables can be accessed via the U.S. Census Bureau (39) and the CDC/ATSDR Social Vulnerability Index (40), and data from the Centers for Medicare and Medicaid Services can be used to identify the geographic locations of hospitals with maternal care services, separated into those able to provide low-acuity and/or high-acuity services (41). It is therefore possible to combine hospital utilization data with demographic, social, and economic data into a maternal healthcare dataset with county-level geographic specificity for the entire country. An added benefit of utilizing these large, public datasets is increased patient privacy and data protection. All data described above are already deidentified and aggregated at levels to prevent re-identification. There can be a potential risk from linking datasets, and protecting patient confidentiality should be the highest priority in any data project, particularly given the need to establish trust among the underrepresented, underserved, and underreported populations these datasets would most benefit. For example, focusing on population-level data (e.g., percent of population or index ratios) would help retain anonymity.

**Table 1:** CCSR pregnancy-related diagnoses.

PRG011	Early or threatened labor
PRG012	Multiple gestation
PRG013	Maternal care related to fetal conditions
PRG014	Polyhydramnios; other amniotic cavity problems
PRG016	Previous C-section
PRG017	Maternal care for pelvic organ abnormality
PRG018	Maternal care related to placenta disorders
PRG019	Diabetes complicating pregnancy/birth
PRG020	Hypertension complicating pregnancy/birth
PRG022	Prolonged pregnancy
PRG023	Complications specified during childbirth
PRG024	Malposition/other labor complications
PRG026	OB-related trauma to perineum and vulva
PRG027	Complication specified during the puerperium
PRG029	Uncomplicated pregnancy/deliver/puerperium

## Discussion

A nation-wide, linked dataset encompassing the maternal health of U3 populations at the geographic level is critically needed to examine specific health issues, identify and address health inequities, and identify geographic barriers to care, as well as to inform the development of effective interventions to improve maternal health outcomes. Utilizing a linked maternal health dataset will enable improvements in research, outreach, and healthcare delivery to understudied, underrepresented, and underreported women according to their geographic residence and proximity to care. Placing the linked data in a publicly available data repository will ensure data sharing and maximize use, which is critical to advance maternal health equity for U3 populations.

Data access and availability have increasingly been a Federal priority with recent advances including the “Holdren Memo” in 2013, the Foundations for Evidence-Based Policymaking Act of 2018 (which includes the Open Government Data Act), and the establishment of data.gov as a data sharing repository. The Confidential Information Protection and Statistical Efficiency Act of 2018 focuses on ensuring confidentiality in shared data, adding to HIPAA protections already in place. These recent legislative and policy advances have had profound impacts on the availability of health data, and they have contributed to all of the datasets described above. These advances have enabled the creation and utilization of the proposed data resource in ways never before possible, making now the perfect time to advance maternal health equity.

A potential limitation in linking large national data sources is that the underlying data are incomplete (i.e., specific sub-populations are not included in the dataset because they do not seek traditional care). One way to examine the extent of missing data is to determine the ratio of routine care visits vs. high acuity visits for each county. A statistically significant increase in high acuity visits for a given county would indicate that routine care data are missing. These instances can be flagged in the dataset and/or adjusted mathematically. Second, data are suppressed when there are fewer than 11 discharges for any given county/CCSR combination or data were not reported. Although this is important to protect patient anonymity, this limitation means that total values are likely to underestimate MMM in the data. Another potential limitation is the large size of the resultant dataset. This could be mitigated by utilizing a server for data storage and MySQL or other software/programming languages designed specifically for large datasets. Finally, another limitation is using the geographic centroid of each county rather than the exact residence location of each perinatal patient. This would result in broader estimates for travel times/distances, particularly in rural areas. However, this is likely necessary given data availability and patient privacy considerations. Similarly, we assume that the overall demographic makeup of a county reflects the demographic makeup of those giving birth within that county, but actual numbers may differ by county and demographic group.

The effort to better understand the geographic barriers for maternal care utilization, particularly among U3 women, is especially significant due to recent increases in MMM and ongoing disparities. Reducing MMM and improving maternal health equity are a national priority. Linking available data from health record databases, demographics, and geographic indicators would enable a county-level investigation into where particular pregnancy-related diagnoses occur, who lives in counties with disparate rates of MMM, and what geographic factors are associated with outcomes. A large, linked dataset allows examination of a wide variety of potentially contributing factors, providing new insights overall, as well as examination of variations in subpopulations or specific geographic regions. However, the true value of a larger dataset is the ability to *compare* contributing factors. For example, race, income, and rurality have all been reported to impact MMM, but these are often overlapping demographics. A large population-level dataset will help researchers disentangle contributing factors and identify the root causes in order to develop more effective interventions. This effort to build a maternal care database will require experience with data management, analysis of big data, medical diagnoses and billing, geographic information systems and geocoding, social determinants of health. Our key recommendations to reduce some of the current challenges and limitations are summarized in Table 2. Despite the challenges, combining available data to better understand geographic barriers to maternal health care will provide a critical resource for research and policy.

**Table 2:** Key recommendations.

• Encourage or require more states to publicly share HCUP data.
• Host workshops where state departments of health can develop common data elements so that publicly available data at the state level are interoperable.
• Establish national-level funding appropriations to implement the unfunded mandates in the Open Data Act.
• Increase grant funding for dataset development.
• Incentivize data sharing following dataset development.

## Data Availability

Publicly available datasets were analyzed in this study. This data can be found here: https://hcup-us.ahrq.gov.
